# Adapting program theory to guide the implementation and evaluation of interventions delivered by advanced practice nurses in Hospital at Home: A programmatic framework for implementation and assessment

**DOI:** 10.1016/j.ijnsa.2025.100444

**Published:** 2025-10-30

**Authors:** Rachid Akrour, Philip Larkin, Henk Verloo

**Affiliations:** a Lausanne University Hospital and University of Lausanne, Institute of Higher Education and Research in Healthcare, Lausanne, Switzerland; b Lausanne University Hospital and University of Lausanne, Site de Cery, Prilly-Lausanne, Switzerland; c School of Health Sciences, HES-SO Valais-Wallis, Sion, Switzerland

**Keywords:** Advanced practice nurses, Hospital at home, Home care services, Chronic disease, Health services accessibility, Outcome assessment, Older adults

## Abstract

This article presents an adaptation of Program Theory framework designed to support the implementation and evaluation of interventions delivered by advanced practice nurses within Hospital at Home models. In response to increasing healthcare demands associated with aging populations and multimorbidity, this framework integrates three interrelated theories; Program Organizational, Service Utilization, and Impact theories to conceptualize and assess advanced practice nurse led care delivery in Hospital at Home settings.

Organizational Theory outlines the structural and functional requirements for integrating advanced practice nurses into Hospital at Home, including role definition, interprofessional collaboration, governance structures, and resource allocation. It emphasizes ways to foster the autonomy of advanced practice nurses, supporting clinical decision-making, and ensuring infrastructure for coordinated care. Service Utilization Theory focuses on the determinants of access, acceptance, and appropriateness of care. It addresses mechanisms for patient referral, eligibility assessment, and care escalation, and highlights the importance of aligning patient needs with the expertise of advanced practice nurses.

The adapted Impact Theory identifies causal pathways linking interventions delivered by advanced practice nurses, such as early assessment, individualized care planning, home visits, therapeutic education, and care coordination to patient and caregiver, for system-level outcomes. These outcomes include reduced hospital admissions and readmissions, fewer emergency department visits, lower nursing home placement rates, and improved functional status, quality of life, and patient satisfaction. The Program Theory framework also supports the evaluation of caregiver burden and the effectiveness of self-management support including health literacy.

Applied in the context of a French-speaking canton in Switzerland, where Hospital at Home services remain underdeveloped and advanced practice nurses are not integrated into these services, this framework provides a structured and theory-driven approach to guide the operationalization and evaluation of their interventions. It establishes a basis for the measurement of outcomes across care processes, individual experiences, and health system impacts. By aligning intervention components with expected outcomes, this approach addresses the complexity of Hospital at Home and the multidimensional contribution of advanced practice nurses, offering a foundation for future implementation and research.

**What is already known about the topic?**

• Hospital at Home is an effective alternative to inpatient care for selected patients, improving outcomes and satisfaction.
• Advanced practice nurses play a key role in managing complex conditions, especially in older populations.
• Program Theory provides a structured method to evaluate complex healthcare interventions.

**What this article adds?**

• Adapts Program Theory to guide the implementation and evaluation of advanced practice nurse-led care in Hospital at Home settings.
• Introduces a context-specific framework for Hospital at Home development in a decentralized Swiss healthcare system.
• Identifies clear outcome measures to assess the contribution of advanced practice nurses at patient, caregiver, and system levels.

## 1 Introduction

The demographic shift toward an aging population with multiple chronic morbidities is reshaping healthcare priorities (Levi et al., 2019). Overall, nearly 80 % of people aged 65 and over live with a chronic illness, of which >68 % live with two or more comorbidities (Ashraf et al., 2024), a trend which is likely to increase. The global proportion of people aged 60 will reach 2.1 billion, with the proportion of people aged 80 and over expected to triple between 2020 and 2050 reaching 426 million (United Nations Department of Economic and Social Affairs, Population Division, 2022). Moreover, older people are more likely to be hospitalized (Chang et al., 2018), and hospital stays for multimorbid older adults are strongly associated with adverse events such as nosocomial infections, delirium, falls, functional decline, frailty and early mortality (Richardson, 2006; Sharek et al., 2011; Shepperd et al., 2017; Sprivulis et al., 2006; Vasilevskis et al., 2012). Consistent with this demographic shift, the World Health Organization calls for a reorientation of services toward person-centered, community-based care that maintains functional ability in older age (World Health Organization, 2025).

Hospital at Home is an acute care model that provides hospital-level treatment in a patient’s home or nursing home. It replaces hospital inpatient care by bringing staff, equipment, technology, and medications to the home. The goal is to improve outcomes and safely deliver hospital-level care outside the hospital (World Hospital at Home Community, 2024). Hospital at Home reduces the length of hospital stays by enabling the transfer of patients from traditional inpatient settings to Hospital at Home care. It can potentially serve as a complete substitute for inpatient care to avoid hospital admission (Gonçalves-Bradley et al., 2017; Shepperd et al., 2016; Shepperd and Iliffe, 1998). The interventions provided by Hospital at Home aim to prevent adverse effects associated with hospitalization while enhancing patient and healthcare professional satisfaction and reducing healthcare costs (Leong et al., 2021). A recent umbrella review concluded that Hospital at Home is a safe and effective alternative for suitable patients requiring hospital-level care at home, demonstrating similar or improved clinical outcomes, reduced length of stay, and increased patient satisfaction (World Hospital at Home Community, 2024). Hospital at Home is suitable for the increasing complexity of care and the pressures on health systems (Arsenault-Lapierre et al., 2021; Levine et al., 2020). However, no single model of Hospital at Home can fit every patient across all healthcare systems. Therefore, it is essential to adapt and promote Hospital at Home models tailored to each health system (Leff et al., 2022, 2000). Given this heterogeneity and the multicomponent nature of Hospital at Home, a coherent theoretical lens is needed to specify expected causal pathways and support comparable evaluation across settings.

The present paper aims to explore how Program Theory can be applied to support the development of a model of care led by advanced practice nurses in Hospital at Home. Specifically, it seeks to identify and address the methodological and operational challenges involved in adapting Program Theory to conceptualize, implement, and evaluate the contribution of advanced practice nurses to Hospital at Home services, and more specifically in the context of a French-speaking canton in Switzerland.

## 2 Program theory as a framework for Hospital at Home

The Hospital at Home model was first evaluated in the United Kingdom in the late 1970s (Pandit et al., 2024). In the United States, a prospective study conducted in the late 1990s among older adults demonstrated the safety and effectiveness of Hospital at Home as an alternative to conventional inpatient care for selected acute medical conditions (Leff et al., 1999). Despite its longevity, Hospital at Home in the United States has evolved with considerable heterogeneity in care delivery, largely due to the absence of formal national guidelines (Levine et al., 2025). A consensus-based national standard was only recently established to provide a unified framework for Hospital at Home practice and to enable consistent evaluation of program performance (Levine et al., 2025). Internationally, while Hospital at Home has been adopted in several countries a comprehensive framework that captures the complexity and technological demands of these services has yet to be developed (Denecke et al., 2024).

The complexity of healthcare interventions necessitates robust evaluation approaches (Rogers, 2008). Several frameworks can guide the evaluation of complex service models such as Hospital at Home. The Consolidated Framework for Implementation Research (CFIR) is well suited to identifying multilevel barriers, facilitators, and contextual influences on uptake (Damschroder et al., 2009). The Reach, Effectiveness, Adoption, Implementation, and Maintenance (RE-AIM) framework helps structure assessment and reporting of population impact and sustainability across those five dimensions (Glasgow et al., 2019; Holtrop et al., 2021). For the role of advanced practice nurses, the Participatory Evidence based Patient focused Process for Advanced practice nursing (PEPPA) offers an evidence-informed, participatory process for design, implementation, and evaluation that complements rather than replaces causal modelling (Bryant-Lukosius et al., 2016). Taken together, these considerations justify using Program Theory as the central analytic approach to specify and test how advanced practice nurse activities, situated in defined contexts, activate mechanisms that generate patient, caregiver, and system outcomes. Such mechanisms should also be consistent with theory-driven and realist evaluation principles that formalize testable context-mechanism-outcome propositions (Chen, 1990; Weiss, 1997).

Program Theory offers a structured approach to assess whether an intervention's components are logically aligned with its intended outcomes, and has increasingly been recognized as rigorous evaluation method (Brousselle and Champagne, 2011; Rogers, 2008). It provides insight not only into whether an intervention works, but critically, into how and why specific outcomes are achieved (Brousselle and Champagne, 2011). This becomes especially relevant in the context of integrated and person-centered care, where interventions are rarely linear. Healthcare models such as Hospital at Home are often characterized by numerous interdependent elements, necessitating sophisticated evaluative frameworks. Therefore, Program Theory offers an appropriate analytical framework to address both complex structures and dynamic interactions (Rogers, 2008). In particular, realist evaluation, a branch of Program Theory grounded in critical realism, has proven useful for evaluating integrated care interventions, as it explores what works, for whom, under what conditions, and through what mechanisms (Smeets et al., 2022). Additionally, Program Theory facilitates the analysis of differential intervention effects across population subgroups. It supports the exploration of how contextual variables such as socioeconomic status, influence program outcomes (Maden et al., 2017).

Chen et al. (2024) proposed a Program Theory as a model adapted to the complex medical-social interventions of Hospital at Home interventions (Chen et al., 2024). They articulated Program Theory through three interrelated theories: Program Organizational, Service Utilization, and Impact theories. Organizational Theory provides a comprehensive framework for the effective delivery of Hospital at Home services by addressing structural, procedural, and contextual elements required for home-based care. It emphasizes resource management through establishing governance structures, aligning services with local needs, integrating telehealth, and forming interdisciplinary teams that incorporate an advanced skill mix. Care delivery is centered on coordinated, multidisciplinary treatment and active collaboration with patients and caregivers, ensuring both acute and holistic care at home. It supports shared workflows, regular team meetings, integrated electronic records, and minimal disruption to patient home environments (Chen et al., 2024).

Service Utilization Theory describes the patient pathway within the Hospital at Home model (Chen et al., 2024). According to this theory, patients are first referred to Hospital at Home and assessed by the Hospital at Home team to evaluate both patient and caregiver eligibility (Chen et al., 2024). Those who are deemed eligible are enrolled and receive acute care at home or in a nursing home. Service Utilization Theory also considers patients who are not referred, not assessed, found ineligible and redirected, or patients who decline participation. Additionally, it accounts for care escalation, including emergency interventions or hospital admission and readmission, which represent critical outcomes in evaluating service use (Chen et al., 2024).

The Impact Theory of Hospital at Home describes the causal pathways through which key components of Hospital at Home services produce patient and system-level outcomes (Chen et al., 2024). It suggests that early and appropriate patient identification, combined with timely, individualized, and acute care at home, mitigates hospital-associated risks. This contributes to faster recovery and supports the preservation of physical, functional, and psychosocial well-being. The theory highlights the role of targeted education and support in enhancing patient and caregiver capacity through improved knowledge, confidence, and adherence. Frequent home visits tailored to patient needs, alongside rapid access to backup services, reduce caregiver burden and reinforce their ability to deliver care. Relational continuity, facilitated by stable care teams and seamless transitions, further enhances patient and caregiver confidence. Collectively, these elements are theorized to reduce hospital (re)admissions, emergency department use, and long-term care placement, while improving satisfaction, lowering costs, and supporting the long-term viability of Hospital at Home services (Chen et al., 2024).

## 3 Hospital at Home an emergent solution in Switzerland

The Swiss healthcare system is unique in its decentralized structure, where healthcare responsibilities are shared between the federal government, cantons, and municipalities (Filliettaz et al., 2021). This multilevel organization leads to a degree of heterogeneity in health policy implementation, which can contribute to fragmentation and regional inequalities in access and services (Monod et al., 2024).

The Hospital at Home model is an innovative and rapidly expanding approach in Switzerland, with several initiatives underway in cities such as Zurich (Hospital at Home AG, 2025), Basel (Klinik Arlesheim, 2025), and Bern (Bern University of Applied Sciences, 2025). Despite the introduction of a minimum dataset to support standardization of care delivery, further work is needed to evaluate safety, efficiency, satisfaction, and cost-effectiveness of Hospital at Home services across the country (Swiss Hospital at Home Society, 2024). Notably, the French-speaking cantons currently lack an operational Hospital at Home program, and none of the existing models in Switzerland explicitly integrate the role of advanced practice nurses.

## 4 The role of advanced practice nurse in Hospital at Home

Advanced practice nursing is an umbrella term encompassing higher levels of nursing practice to address complex healthcare needs. It represents an advanced scope of care that leverages in-depth nursing knowledge and expertise to meet the health requirements of individuals, families, groups, communities, and populations (DiCenso et al., 2010). Internationally, there is consensus that the competencies and knowledge required for the role of advanced practice nurses should be acquired through education at the level of Masters degree (International Council of Nurses, 2020). The advanced practice nurse designation generally includes clinical nurse specialists and nurse practitioners. Both roles reflect advanced decision-making skills, expert knowledge, and clinical competencies essential for delivering specialized and comprehensive care (International Council of Nurses, 2020).

Advanced practice nurses are central in complex care models across various dimensions, such as planning and coordination, primary and secondary disease prevention, leadership, patient follow-up, advocacy, research, and translating evidence into practice (Kleinpell et al., 2024)**.** These dimensions are particularly true for older adults, whose condition is closely linked with aging and frailty and is characterized by multiple complex chronic conditions, frequent hospital admissions and high utilization of health services, polypharmacy, and reduced personal autonomy (Morilla-Herrera et al., 2016). According to the American Association of Nurse Practitioners, nurse practitioners are licensed, independent practitioners who practice both autonomously and in collaboration. They can provide acute care across settings, including care delivered in the home. This substantiates both the organizational requirement for professional autonomy and the clinical delivery assumptions articulated in the Impact domain of this framework (American Association of Nurse Practitioners, 2022).

Policy resources from the American Association of Nurse Practitioners articulate full practice authority as an evidence-based approach enabling nurse practitioners to evaluate, diagnose, order and interpret tests, and initiate and manage treatments without unnecessary restrictions, aligning with the autonomy required for hospital-level care at home (American Association of Nurse Practitioners, 2022). Building on the scope outlined above, compared with generalist nurses, the added value derives from graduate-level diagnostic reasoning, prescribing authority, and autonomous decision-making, which allow advanced practice nurses to act as first-contact clinicians for acute presentations, manage higher-risk complexity, and lead cross-setting coordination without avoidable delays (American Association of Nurse Practitioners, 2022; International Council of Nurses, 2020).

In operational terms for Hospital at Home, advanced practice nurses conduct comprehensive advanced clinical assessments, order and interpret diagnostics, formulate differential diagnoses and management plans, initiate and titrate treatments within their prescriptive authority, and lead protocolized escalation and interprofessional coordination when predefined thresholds are met, in collaboration with physicians as needed. These functions reflect graduate-level preparation and regulated scope described in international guidance and professional standards (American Association of Nurse Practitioners, 2022; International Council of Nurses, 2020).

Evidence from a recent scoping review indicates that nurse-led models of care demonstrated significant positive outcomes across healthcare. These include reduced hospital length of stay, cost savings, enhanced chronic disease management and prevention, improved clinical indicators, increased screening, and greater healthcare accessibility with high patient and provider acceptance. While the majority of findings are supportive, isolated studies report limited or no benefit in specific settings, underscoring the need for contextual consideration in implementation (Beks et al., 2023). Consistent with these trends, a case-control evaluation of a Hospital at Home model bundled with 30-day transitional care reported a shorter acute length of stay (3.2 versus 5.5 days; difference −2.3 days), fewer 30-day readmissions (8.6 % versus 15.6 %), fewer emergency department revisits (5.8 % versus 11.7 %), fewer admissions to skilled-nursing facilities (1.7 % versus 10.4 %), and higher overall ratings of care (68.8 % versus 45.3 %) for patients receiving Hospital at Home services (Federman et al., 2018). In an observational cohort study of community management for acute exacerbations of chronic obstructive pulmonary disease staffed by nurse practitioners, no deaths occurred during the exacerbation episodes studied. Hospitalization rates fell by 20 % within the service’s catchment, and both hospital and home-managed groups improved on the St George’s Respiratory Questionnaire activity domain and the Transitional Dyspnea Index from exacerbation to recovery (Ansari et al., 2009). These results are concordant with broader syntheses indicating that advanced practice nurses achieve clinical outcomes comparable to physician-led models, while improving access and efficiency, capabilities that are central to safe Hospital at Home delivery (Htay and Whitehead, 2021; Laurant et al., 2018; Newhouse et al., 2011).

Contemporary research increasingly supports the effectiveness of advanced practice nurses in managing complex conditions. Evidence supports the role of advanced practice nurses in enhancing continuity of care and clinical outcomes, improving the safety and quality of care, reducing costs, and increasing patient, family, and colleague satisfaction in diverse healthcare settings and populations (Kilpatrick et al., 2024; Morilla-Herrera et al., 2016). Moreover, the responsibilities of advanced practice nurses extend beyond traditional patient care, encompassing health promotion, disease prevention, and therapeutic management, particularly for individuals with chronic and complex conditions (Lecocq et al., 2015). Advanced practice nurses operate with high professional autonomy, engaging in case management, advanced assessment, diagnosis, decision-making, and consultancy (Morilla-Herrera et al., 2016). They also contribute significantly to developing health programs and ensuring the coordination of patient care across multiple providers (Morilla-Herrera et al., 2016). For older adults, two distinct models of advanced practice nursing have been identified: a disease-oriented approach focusing on risk management through evidence-based interventions and a generalist model aimed at fostering patient autonomy (Morilla-Herrera et al., 2016). Both models demonstrated positive outcomes, particularly in long-term care settings, where the expertise of advanced practice nurses in educational interventions and multidimensional assessments enhances continuity of care (Morilla-Herrera et al., 2016). In a multicenter randomized trial of admission-avoidance by Hospital at Home with comprehensive geriatric assessment (a service that included nurse practitioners), the proportion living at home at six months was similar to inpatient care (78.6 % versus 75.3 %), mortality did not differ (16.9 % versus 17.7 %), and admissions to long-term residential care were lower with Hospital at Home interventions (5.7 % versus 8.7 %, risk ratio 0.58, 95 % confidence interval 0.45–0.76) (Shepperd et al., 2021).

Given the complexity of Hospital at Home intervention models, it is crucial to understand where nursing makes a difference in patient care and impacts positively on the quality of care provided (Oner et al., 2020). Thus, a structured and comprehensive approach is necessary to evaluate advanced practice nurses’ roles effectively (Younger, 2020). Bryant‐Lukosius and DiCenso (2016) suggest that the evaluation should encompass structure, process, and outcomes. Structure refers to the resources, organizational environment, and characteristics of the advanced practice nurse role. Process refers to the delivery of services across clinical practice, education, research, and leadership, and outcome focuses on measurable results such as patient safety, efficacy, satisfaction, cost-effectiveness, and role transfer (Bryant-Lukosius et al., 2016).

However, improving healthcare quality within complex systems presents significant challenges. Many current initiatives rely on rigid, linear strategies that fail to address healthcare's dynamic and multidimensional nature. To address this, quality improvement for advanced practice nurses should favor flexible, adaptive approaches that respond effectively to evolving healthcare needs while ensuring meaningful and measurable improvements (Younger, 2020). Accordingly, defining and measuring outcomes is essential to demonstrate the effects of these advanced practice nurse activities on patients, caregivers, and the health system.

## 5 Outcomes related to interventions delivered by advanced practice nurses

Within a multidisciplinary team, the unique contribution of advanced practice nurses can be difficult to determine (Morilla-Herrera et al., 2016). However, the coordinated approach of advanced practice nurses is critical to delivering holistic care. In disease-specific conditions, such as late-stage cancer, advanced practice nurses play a vital role in managing the holistic needs of the patient (McCorkle et al., 2015). Moreover, evaluating the impact of advanced practice nurses on patient outcomes and care quality is essential for performance assessment, especially as their involvement in patient care management expands across various healthcare settings, including hospitals, outpatient facilities, and community practices (Kleinpell et al., 2024). Research has demonstrated that advanced practice nurses influence a range of measurable outcomes (Bryant-Lukosius et al., 2015). For instance, in long-term settings, care delivered by advanced practice nurses is associated with reduced readmissions and increased patient and caregiver satisfaction (Morilla-Herrera et al., 2016). Similarly, advanced practice nurse involvement for patients with heart failure implies a reduction in the number of hospital readmissions (Ordóñez-Piedra et al., 2021). Furthermore, studies on clinical nurse specialists showed that care delivered by clinical nurse specialists significantly reduced patient mortality and improved treatment adherence and patient satisfaction (Bryant-Lukosius et al., 2015).

Evidence syntheses examining interventions provided by advanced practice nurses across diverse healthcare contexts identified consistent improvements in access to care, continuity, and quality. Access is enhanced through timely assessment by advanced practice nurses (Beks et al., 2023; Kilpatrick et al., 2024), while individualized advanced care planning supports personalized, coordinated interventions (Morilla-Herrera et al., 2016; Rodríguez-García et al., 2025). Advanced practice nurses also facilitate coordination, reinforcing integrated care pathways (Kilpatrick et al., 2024). Home visits, therapeutic education, and support for self-management have shown beneficial effects on literacy, engagement, and adherence (Woo et al., 2024). These interventions are linked to reduced adverse events, shorter care episodes, improved nutritional and functional status, better quality of life, and decreased burden on caregivers (Audet et al., 2021; Ordóñez-Piedra et al., 2021). Importantly, care delivered by advanced practice nurses contributes to lower rates of hospital and emergency admissions, fewer nursing home placements, and reduced mortality in several studies (Htay and Whitehead, 2021; Kilpatrick et al., 2024; Morilla-Herrera et al., 2016). These findings emphasize the relevance of outcome-based evaluation to fully understand and support the role of advanced practice nurses in complex care delivery models.

This said, despite growing evidence on the effectiveness of nurse-led interventions administered by advanced practice nurses, data specifically evaluating their outcomes within Hospital at Home remain limited and underexplored (Akrour et al., 2025).

## 6 Adaptation of program theory to the advanced practice nurse role in Hospital at Home and its evaluation

Drawing upon the framework presented by Chen et al. (2024), Program Theory offers salient perspectives for analyzing the integration and function of advanced practice nurses within the Hospital at Home. In the adapted framework, advanced practice nurse-specific elements are introduced at three levels. Within Organizational Theory, role delineation, diagnostic and prescribing authority, and protocolized escalation are specified. Within Service Utilization Theory, referral, eligibility, acceptance, and appropriateness criteria are defined in relation to advanced practice nurse scope. Within Impact Theory, the content of advanced practice nurse-delivered processes is articulated, direct and indirect mechanisms are identified, and a cross-cutting patient-reported outcomes and experiences measurement layer is added.

## 7 Organizational theory and the role of advanced practice nurses in Hospital at Home

Organizational Theory defines the structural and functional requisites for the effective deployment of advanced practice nurses within Hospital at Home settings.

**Structure:** The organizational architecture of Hospital at Home services must be configured to foster the autonomy advanced practice nurses and support collaborative practice. This requires clear role definitions, responsibilities, and lines of reporting so that advanced clinical competencies can be exercised within a multidisciplinary context. Robust communication channels and information management systems are indispensable to ensure seamless information exchange and the effective coordination of advanced practice nurse care activities. Furthermore, allocating adequate resources, encompassing staffing levels, technological infrastructure, and access to ancillary services, is paramount to facilitate the delivery of high-quality care by advanced practice nurses. Under this adaptation, organizational structures include advanced practice nurse privileges for ordering and interpreting tests, prescribing within scope, named escalation protocols with collaborating physicians, and documentation pathways that recognize the advanced practice nurse as the first-contact clinician where appropriate.

**Function:** Organizational Theory also emphasizes leadership and governance that support the integration of the advanced practice nurse role. This includes proactive leadership that champions the role of advanced practice nurses and cultivates a culture of interprofessional collaboration, and formalized policies and clinical protocols that delineate the scope of the advanced practice nurse role, enabling advanced assessments, diagnostic reasoning, and therapeutic interventions within defined authorization. Opportunities for continuous professional development are vital to maintain the expertise required for advanced practice nursing and to ensure adherence to evidence-based practice guidelines. Performance evaluation systems should provide appropriate recognition and reward for the contributions of advanced practice nurses, thereby supporting job satisfaction and professional retention.

## 8 Service utilization theory and the advanced practice nurses’ role in Hospital at Home

In this context, Service Utilization Theory focuses on the determinants and patterns of Hospital at Home service use, specifically regarding the expertise of advanced practice nurses. Service Utilization Theory encompasses referral pathways, eligibility assessment, acceptance, and escalation mechanisms. Within the adapted framework, referral and eligibility routes direct appropriate cases to an advanced practice nurse-led first-contact assessment. Acceptance factors incorporate patient and caregiver understanding of the advanced practice nurse role and scope. Appropriateness criteria align clinical indications with advanced practice nurse competencies using evidence-based guidelines and predefined escalation thresholds.

**Access:** The facilitation of patient access to services delivered by advanced practice nurses within the Hospital at Home model is of paramount importance. This entails the establishment of transparent referral pathways, responsive triage mechanisms, and flexible scheduling protocols that are tailored to patient-specific needs. Reducing barriers, such as geography and preferences, optimizes effective use of services delivered by advanced practice nurses.

**Acceptance:** Patient and caregiver acceptance of the role of advanced practice nurses is a critical determinant of Hospital at Home program success. This necessitates clear communication strategies and targeted education that build a shared understanding of the qualifications of advanced practice nurses and the scope of their practice. The cultivation of trust and rapport between advanced practice nurses, patients, and their caregivers is essential to increase acceptance and sustain a collaborative care environment.

**Appropriateness:** Ensuring the judicious utilization of services delivered by advanced practice nurses involves the alignment of patient clinical needs with the expertise of advanced practice nurses. This requires the development and implementation of evidence-based clinical guidelines and protocols that clearly define the target populations and clinical scenarios in which involvement of advanced practice nurses is most effective. Systematic review and audit of utilization patterns for advanced practice nurses are essential to ensure the effective and efficient deployment of their advanced skills.

## 9 Impact theory and the role of advanced practice nurses in Hospital at Home

Consistent with the boundaries above, Impact Theory covers the delivery and content of interventions by advanced practice nurses as well as their effects on patient, caregiver, and system outcomes.

**Fig. 1:**
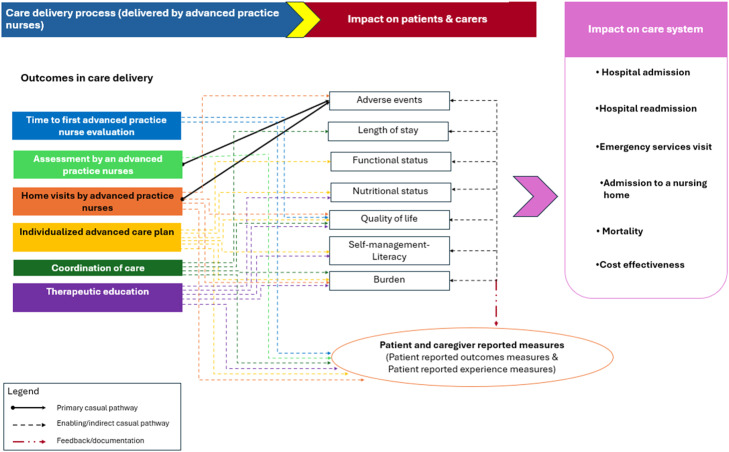
Chen et al. (2024) Impact Theory adapted to the interventions of advanced practice nurses in Hospital at Home.

Fig. 1 presents an adapted Impact Theory by Chen et al. (2024) illustrating the role of care delivered by advanced practice nurses and its subsequent effects. The process starts with the assessment conducted by advanced practice nurses, leading to an individualized advanced nurse-led care plan and coordination of care. Home visits are a key component and influence time to the first advanced practice nurse evaluation. These interventions are theorized to influence patients and caregivers through several pathways, affecting adverse events, length of stay, functional and nutritional status, health-related quality of life, therapeutic education, self-management literacy, and caregiver burden. Within the adapted Impact Theory, advanced practice nursing interventions contribute to outcomes through both primary and enabling mechanisms. Timely first evaluation and advanced clinical assessment act as direct determinants of safety by reducing adverse events and ensuring appropriate utilization (Ansari et al., 2009; Imhof et al., 2012). In particular, systematic assessment by advanced practice nurses provides an entry point for identifying risks early, which directly reduces adverse events and indirectly supports improvements in quality of life and satisfaction for patients and caregivers (Imhof et al., 2012). Individualized advanced care plans generate direct effects on functional status, nutritional well-being, adherence, and quality of life by structuring patient-centered care pathways (Isautier et al., 2019; Morilla-Herrera et al., 2016; Rodríguez-García et al., 2025). Home visits provide direct support to safety and continuity through ongoing monitoring and responsiveness to acute changes (Ansari et al., 2009; Bryant-Lukosius et al., 2015). Coordination of care and therapeutic education operate as enabling mechanisms, strengthening relational quality, self-management literacy, and adherence, and thereby mediating effects across several domains (Audet et al., 2021; Kripalani et al., 2009; Woo et al., 2024).

This classification of links as primary or enabling follows realist evaluation principles, which distinguish proximal generative mechanisms from context-contingent, mediated effects, thereby yielding testable propositions and reducing construct conflation (Chen, 1990; Moore et al., 2015; Weiss, 1997). Ultimately, these pathways contribute to improved satisfaction for patients and caregivers, measured through standardized outcome instruments. Patient-reported outcome measures (PROMs) capture health status, functional capacity, and quality of life (Bull et al., 2022; Snowdon et al., 2023), while patient-reported experience measures (PREMs) reflect evaluations of communication, coordination, and timeliness of care (Jamieson Gilmore et al., 2023; Virdun et al., 2023). Within the perspective of Impact Theory, patient-reported outcome measures capture the direct results of care on patients, such as symptoms, functional capacity, or quality of life. In contrast, patient-reported experience measures capture how patients and caregivers perceive the processes of care, including communication, coordination, and timeliness. These reported experiences can influence or shape the effects of care, either by mediating or moderating how clinical interventions translate into outcomes (Bonsel et al., 2024; Casaca et al., 2023).

At the system level, advanced practice nursing embedded in multidisciplinary teams contributes to shorter stays, fewer readmissions, and reduced emergency use, which are recognized as advanced practice nursing–sensitive indicators of efficiency and continuity (Kleinpell et al., 2024; Morilla-Herrera et al., 2016; Woo et al., 2024). The adapted model distinguishes activities that function as direct mechanisms from those that act through enabling conditions, while positioning patient-reported measures as a cross-cutting layer for monitoring and continuous improvement.

Table 1 summarizes the assignment of each construct to its primary locus (Program Organizational, Service Utilization, Impact theories) and highlights the advanced practice nurse specific adaptations in organizational preconditions, utilization pathways, and mechanism designation.

**Table 1:** Mapping of core constructs to Organizational, Service Utilization, and Impact theories.

**Core construct**	**Organizational theory (structural & enabling conditions)**	**Service Utilization theory (access, acceptance, appropriateness)**	**Impact theory (intervention delivery & effects)**	**Primary locus**
Governance & role definition	Establishes the advanced practice nurse role, accountability lines, decision rights, and authorization protocols	—	Shapes permissible scope of interventions and decision-making in practice	Organizational Theory
Resources & infrastructure	Provides staffing levels, training budget, digital tools, and access to ancillary services	—	Determines feasibility, intensity, and reliability of interventions and follow-up	Organizational Theory
Communication & information systems	Implements communication pathways and shared records to support coordination	—	Enables timely care planning, monitoring, and information exchange during episodes	Organizational Theory
Referral pathways	Sets policy support and interfaces with referrers	Designs and operates clear pathways that trigger enrollment	Affects timeliness of assessment and initiation of care	Service Utilization Theory
Eligibility & triage	Provides criteria governance and safety oversight	Applies criteria to match patients to the model, prioritizes scheduling	Influences when and how interventions commence	Service Utilization Theory
Acceptance (patients/caregivers)	Supports education materials and engagement strategy	Builds understanding and trust, addresses preferences and concerns	Higher acceptance improves adherence to plans and education uptake	Service Utilization Theory
Appropriateness (care-need fit)	Endorses evidence-based protocols and audit expectations	Aligns patients’ needs with the expertise of advanced practice nurses	Appropriate matching improves clinical and experiential outcomes	Service Utilization Theory
Escalation & transitions	Defines escalation policies, thresholds, and handover agreements	Activates escalation/transfer mechanisms when criteria are met	Ensures continuity and safety during changes in acuity or setting	Service Utilization Theory
Individualized care planning	Provides templates/standards for documentation	—	Advanced practice nurses develop and adapt patient-specific plans	Impact Theory
Intervention delivery (visits, coordination, therapeutic education)	Ensures role authorization, supplies, and logistics	—	Advanced practice nurses conduct assessments, provide education, coordinate care	Impact Theory
Monitoring & reassessment	Supports quality routines and data capture	—	Advanced practice nurses track progress and adjust plans	Impact Theory
Outcomes: patient and caregiver, system	Enables measurement systems and reporting	—	Reflect effects of the intervention pathways over time	Impact Theory

Positioning patient-reported outcome and patient-reported experience measures through cross-cutting feedback and documentation reflects their status as measurement instruments that inform monitoring and iterative improvement rather than as outcomes in themselves, consistent with established quality improvement and complex intervention guidance (Moore et al., 2015; Organization for economic co-operation and development, 2019; Skivington et al., 2021). Interdependencies among safety and utilization, function and quality, and experience and engagement are treated as indirect and often bidirectional links, while a feedback/documentation layer represented by dotted connections from each cluster to patient reported outcome and patient reported experience measures supports learning and model refinement. System-level impact includes hospital admissions and readmissions, emergency department use, nursing-home placement, mortality, and costs and resource use. When quality-adjusted outcomes are available, cost-effectiveness can be estimated. Including costs and resource use captures the immediate budget effects of safety and utilization performance and the longer-term consequences mediated by functional trajectories, consistent with guidance for complex interventions (Moore et al., 2015; Skivington et al., 2021).

This final section sets out the evaluation approach for advanced practice nurse interventions, linking the adapted theory to measurable outcomes and study procedures.

To operationalize the adapted Impact Theory in the context of care administered by advanced practice nurses within Hospital at Home, this paper proposes a framework for evaluating a model of interventions conducted by advanced practice nurses in a French speaking canton of Switzerland, where Hospital at Home services are still in the early stages of development. While the diagram illustrates the theoretical causal pathways through which advanced practice nurses influence patient and system outcomes, this evaluation approach has not yet been implemented. The proposed framework is intended to guide development and evaluation of the advanced practice nurse care model within Hospital at Home.

This adapted Impact Theory from Chen et al. (2024) maps the expected clinical, patient, and system-level outcomes. These outcomes in Table 2 are informed by prior empirical literature (Audet et al., 2021; Beks et al., 2023; Bryant-Lukosius et al., 2015; Htay and Whitehead, 2021; Kilpatrick et al., 2024; Morilla-Herrera et al., 2016; Ordóñez-Piedra et al., 2021; Rodríguez-García et al., 2025; Woo et al., 2024), and reflect the multidimensional impact of advanced practice nurses in complex care models. To operationalize measurement, a non-exhaustive set of validated instruments can be used, for example EuroQol Five-Dimension five-Level (EQ-5D-5L) for health-related quality of life, the Barthel Index for functional status, and the Care Transitions Measure for continuity and experience, with locally adapted versions employed where appropriate. Data collection will rely on standardized tools administered at predefined intervals, ensuring consistency and comparability across patients. Validation is addressed using instruments with established psychometric properties, with adaptations made only to align with local language and context.

**Table 2:** Outcomes of evaluation of advanced practice nursing interventions in Hospital at Home.

**Outcomes**	**Measures**	**Evaluation** **(Tools/scales)**	**Descriptions**
Outcomes in the Process of Care	Time to first advanced practice nurses evaluation: access to advanced practice nurse care (Beks et al., 2023; Kleinpell et al., 2024; Woo et al., 2024)	Time to first advanced practice nurse home visit (days) (Administrative/clinical records)	Measures the efficiency of initiating Hospital at Home care with advanced practice nurse involvement and access to Hospital at Home care.
	Individualized advanced care plan (Morilla-Herrera et al., 2016; Rodríguez-García et al., 2025)	Proportion of patients with an individualized advanced care plan documented within 1 day of Hospital at Home admission: Assesses the implementation of patient-centered planning. (Administrative/clinical records)	Assesses the implementation of patient-centered planning.
	Coordination of care (Kilpatrick et al., 2024; Rodríguez-García et al., 2025)	Number of interprofessional care coordination activities per patient episode (Relational Coordination Survey (Hoffer Gittell, 2002))	Quantifies the advanced practice nurse role in linking different services
	Advanced practice nurse visits at home (Kilpatrick et al., 2024)	Frequency and duration of advanced practice nurse home visits per patient episode (number and time) (Administrative/clinical records)	Measures the intensity of advanced practice nurse contact.
	Therapeutic education (Kilpatrick et al., 2024; Rodríguez-García et al., 2025; Woo et al., 2024)	Change in patient adherence to therapeutic recommendations during the Hospital at Home episode (Adherence to Refills and Medications Scale (ARMS) (Kripalani et al., 2009))	Advanced practice nurses provide education to patients and caregivers, which is linked to improved self-management
	Self-management (Literacy) (Kilpatrick et al., 2024; Rodríguez-García et al., 2025; Woo et al., 2024)	Change in patient self-management literacy score (using a validated tool) during the Hospital at Home episode (Health Literacy Questionnaire (Osborne et al., 2013), MEDication literacy assessment of geriatric patients and informal caregivers (MED-fLAG) (Gentizon et al., 2022))	Measures the impact on patient empowerment.
Outcomes for Patients and Caregivers	Adverse events (Kilpatrick et al., 2024; Morilla-Herrera et al., 2016; Rodríguez-García et al., 2025)	Number of adverse events (e.g., falls, medication errors) during the Hospital at Home episode (Administrative/clinical records)	Measures safety outcomes
	Length of Stay (Audet et al., 2021; Bryant-Lukosius et al., 2015; Kleinpell et al., 2024)	Length of Hospital at Home stay (in days) (Administrative/clinical records)	Captures the duration of home-based acute care
	Functional and nutritional status (Audet et al., 2021; Bryant-Lukosius et al., 2015; Kilpatrick et al., 2024)	Change in functional status (using a validated tool) during and post Hospital at Home episode / Change in nutritional status (using a validated assessment) during the Hospital at Home episode Barthel Index (Hopman-Rock et al., 2018), Timed Up and Go (Exter et al., 2024; Persson et al., 2014), Mini Nutritional Assessment-Short Form (Isautier et al., 2019)	Assesses the impact on physical abilities/ Assesses nutritional well-being.
	Quality of life (Audet et al., 2021; Kilpatrick et al., 2024; Ordóñez-Piedra et al., 2021)	Change in quality-of-life score (using a validated patient reported outcomes measures) during and post Hospital at Home episode (EuroQol five-Dimension Five-Level (EQ-5D-5 L)) (Feng et al., 2021))	Captures the patient's perspective on their well-being
	Burden of patients and caregivers (Bryant-Lukosius et al., 2015)	Caregiver burden score (using a validated tool) at the start and end of the Hospital at Home episode (Zarit Burden Interview (ZBI-22) (Bianchi et al., 2016), Zarit Burden Interview (ZBI-12) (Gratão et al., 2019))	Measures the impact on caregivers
	Patients and caregivers’ satisfaction (Audet et al., 2021; Htay and Whitehead, 2021; Kleinpell et al., 2024)	Patient and caregiver satisfaction with care by advanced practice nurses in Hospital at Home (using a validated patient reported experience measures) at dischargeccf (Home Health Care Survey of the Consumer Assessment of Healthcare Providers and System (Iovino et al., 2025), Caregivers’ Experience Instrument (IEXPAC CAREGIVERS) (Guilabert et al., 2018)	Assesses experience of patients and caregivers
Outcomes for the Care System	Hospital admission (Audet et al., 2021; Bryant-Lukosius et al., 2015; Rodríguez-García et al., 2025)	Number of hospital admissions from the Hospital at Home cohort during the episode of care (Administrative/clinical records)	Measures the effectiveness of preventing inpatient stays
	Hospital readmission (Audet et al., 2021; Kleinpell et al., 2024; Morilla-Herrera et al., 2016)	30-day and 90-day hospital readmission rates for the Hospital at Home cohort (Administrative/clinical records)	Measures the sustainability of home-based care.
	Emergency services visit (Audet et al., 2021; Bryant-Lukosius et al., 2015; Kilpatrick et al., 2024)	Number of emergency department visits from the Hospital at Home cohort during and within 30 days post-discharge (Administrative/clinical records)	Measures the impact on acute care utilization
	Admission to a nursing home (Htay and Whitehead, 2021)	Rate of admission to a nursing home within 90 days post Hospital at Home discharge (Administrative/clinical records)	Measures the impact on long-term care placement
	Mortality (Morilla-Herrera et al., 2016; Ordóñez-Piedra et al., 2021; Woo et al., 2024)	Mortality rate of the Hospital at Home cohort during and within 30/90 days post-discharge (Administrative/clinical records)	Measures the impact on survival

Analysis will focus on linking indicators to the hypothesized causal pathways, enabling both outcome evaluation and feedback for continuous model refinement, while remaining consistent with international standards. As shown in Table 2, outcomes are explicitly positioned within the adapted Impact Theory framework, ensuring that measurement is not only descriptive but also tests the theorized causal pathways between advanced practice nurse activities, mechanisms, and observed results. The proposed framework, therefore, aims to guide the structured and context-specific evaluation of advanced practice nurse interventions in Hospital at Home, supporting their integration and effectiveness within the Swiss healthcare system.

## 10 Conclusion

Evaluating the specific contribution of advanced practice nurses within the Hospital at Home model presents a challenge due to the complexity of multidisciplinary care. However, this paper proposes an adaptation of Chen et al. (2024) Program Theory to support the implementation and evaluation of interventions delivered by advanced practice nurses in Hospital at Home settings. By integrating organizational, utilization, and impact theories, the framework clarifies how advanced practice nurse led care contributes to improved patient, caregiver, and system outcomes. This addresses an existing gap in current Hospital at Home models and is especially relevant where advanced practice nurse roles remain ill-defined, such as in French-speaking regions of Switzerland. The adapted Program Theory provides a structured approach to guide the development, operationalization, and outcome measurement of interventions administered by advanced practice nurses in Hospital at Home. This framework may also be transferable to other decentralized health systems where responsibilities are distributed across regional and local levels, as it explicitly links organizational arrangements with service use and outcomes.

## Authors contribution

RA contributed to the conception and design of the study, data analysis, and drafting of the manuscript. HV and PL contributed to the critical revision of the article for important intellectual content and provided supervision throughout the development of the manuscript. All authors reviewed and approved the final version of the manuscript and agree to be accountable for all aspects of the work.

## Funding

This work received no funds or grants.

## CRediT authorship contribution statement

**Rachid Akrour:** Writing – original draft, Visualization, Validation, Methodology, Formal analysis, Data curation, Conceptualization. **Philip Larkin:** Writing – review & editing, Validation, Supervision, Methodology. **Henk Verloo:** Writing – review & editing, Visualization, Validation, Supervision, Methodology.

## Declaration of competing interest

The authors declare that they have no known financial or personal relationships with any individuals or organizations that could inappropriately influence or bias the content of this work.
